# Women's Volunteering and Voluntary Leadership Positions in Sport—Secondary Analyses of the German Survey on Volunteering

**DOI:** 10.3389/fspor.2022.871907

**Published:** 2022-08-11

**Authors:** Ulrike Burrmann, Stephan Sielschott

**Affiliations:** Institute of Sport Science, Humboldt-Universität zu Berlin, Berlin, Germany

**Keywords:** gender, intersectional analysis, leadership position, voluntary work, volunteering

## Abstract

For decades, the German sports policy mission statement “Sport for All” has been aimed at attracting women to voluntary work in the sports sector. Nevertheless, women are consistently underrepresented in volunteering within sports organizations and especially on boards. One-dimensional gender analyses that exclude other factors like class and ethnicity cannot, however, adequately describe different modes of disadvantage. In order to analyze the unequal access to volunteering and leadership positions in sport, we refer to inequality theory and intersectional approaches, which include different factors of disadvantage. Our study is based on a quantitative population survey on volunteering in Germany with more than 25,000 respondents conducted in 2014 and 2019. We examine factors and interactions that can predict women's volunteering and leadership in sport. The results show that the proportion of women who volunteer is lower than the proportion of men and that fewer women than men take on leadership positions. The gender differences were not as pronounced in 2019 as they were in 2014. Independent of gender, the likelihood of volunteering increases with higher income, A-levels, no immigration status, marriage and the presence of children in the household. Part-time and marginal employment is more often associated with volunteering among women than among men; however, the likelihood of volunteering decreases more for women than for men when they are not employed at all. Moreover, higher income for women is less likely to be associated with voluntary work than for men while volunteering in other areas has a more positive effect on volunteering in sports for women than for men. Independent of gender, the likelihood of holding a leadership position increases with higher income, with marriage, and decreases with immigration background and with the presence of children in the household. Part-time and marginal employment increase the likelihood of having a leadership position to a greater extent for men than for women. In terms of leadership positions men benefit more than women if there are no children in the household. The results suggest that practical and policy efforts should focus more on improving the conditions for women to gain voluntary leadership positions.

## Introduction

### Relevance and Objective

For decades, the sports policy mission statement “Sport for All” has been aimed in particular at addressing and attracting women to sports activities, as well as to voluntary work in the sports sector (especially in sports clubs) in Germany. “Frauen an die Spitze” (“Women to the top”) was, for example, an initiative of the German Sports Confederation (DSB) and the Federal Ministry for Family Affairs, Senior Citizens, Women and Youth (BMFSFJ) in the early 2000s, and was intended to contribute to the equal participation of women and men in voluntary leadership positions in sports organized by clubs and associations (BMFSFJ, 2004). For a number of years, mentoring programs in sport have been supported by both the German Olympic Sports Confederations (DOSB) and by some member organizations as an instrument of personnel development. The aim is to support qualified young professionals who want a career in organized sport. There is also an expectation, above all, that mentoring programs will increase the proportion of female managers in sports. In 2012, for example, the DOSB launched the mentoring project “To the top with a mixed double!” with the aim of recruiting former female athletes for voluntary or full-time leadership positions in organized sport (DOSB, 2013). A gender quota has been enshrined in the DOSB statutes since 2014. The target quota for individual elections and for members of the DOSB Executive Board is for women and men to each comprise at least 30% of all DOSB bodies (DOSB, 2017; for an overview see Hartmann-Tews, 2019).

Such programs and initiatives to promote women in sport and voluntary work can also be found in other countries and international organizations (Adriaanse, 2017). An example of this is the “Balance in Sports Project” from 2016, which led to the development of tools to implement gender equality or the project “All in: Towards gender balance in sport” from 2018 to 2019 (Council of Europe, n. d.). At the international level, the United Nations Entity for Gender Equality and the Empowerment of Women (UN Women) “invites members of the sport ecosystem to join the Sports for Generation Equality Initiative to accelerate progress on a set of common principles and aligned objectives that will harness the power of sport in making gender equality a reality in and through sport” (UN Women, 2020, p. 1). The extent to which this can increase the proportion of women volunteering apparently depends on regional, national and organizational acceptance and is still debated (Evans and Pfister, 2021). Baxter et al. (2021) summarize that while women's participation in sport has increased worldwide over the last two decades, the number of women volunteers (e.g., referees, judges) has not increased to the same extent. This also applies in particular to the representation of women in top positions in sports organizations (Kvande, 2007). In Germany, the status is as follows: Only two out of the 16 state sports federations are currently led by a woman. In five out of 58 national sport associations, the voluntary leader is female, and in six associations there is a female/male tandem leadership. A positive development can be seen in the proportion of women in the presidia and executive boards of the state sports federations, seven of which reach the quota of 30%. Just under a quarter of the 58 participating national sport associations have at least 30% women on their voluntary boards. However, there are also 13 associations without women on the executive committee. The average proportion of women is 18% (DOSB, 2021). In 2021, according to the organization's own data, 11 out of 87 member organizations have established a mentoring program. Over the last five years, 17 associations had their own mentoring program (DOSB, 2021). In view of these numbers, it can already be seen as a success that women make up 44.4% of the German Olympic Sports Confederation's Executive Board, presumably due to the gender quota mentioned above (DOSB, 2021).

The sports development report 2017/2018 is based on feedback from clubs and its results show that in 2017 the share of women on sports clubs' boards was 30.7%, which is still substantially below the average female membership per association of 35.9%. Women are most often engaged as secretaries (46.3%) and youth leaders (37.4%), but least often as chairpersons (16.0%) (Breuer and Feiler, 2020).

The underrepresentation of women in volunteer and especially in leadership positions in club sports is not unique to Germany (for an overview see Burton and Leberman, 2017; Elling et al., 2019). The proportion of women on the boards of national sport federations in European Union countries is 14% (Wicker et al., 2020 based on Data from European Institute for Gender Equality). A similar trend was found by Adriaanse (2016) and Tranter et al. (2016). Van der Roest et al. (2017) report that in all ten countries they studied, men are overrepresented relative to women as members and volunteers in community sports clubs. Gender equality is most likely to be found in the Scandinavian countries, while studies from Spain and Poland tend to indicate significantly higher proportions of men (Burton and Leberman, 2017; Lamprecht et al., 2017; Sisjord et al., 2017; Evans and Pfister, 2021).

The consistent and global underrepresentation of women in volunteering within sport organizations and especially on boards could be an indicator of gender inequality. However, one-dimensional gender analyses fall short of adequately describing forms of discrimination, power relations, subject positions and social inequalities in voluntary engagement and leadership positions in sport. Moreover, the focus is often on sports associations and on leadership positions (Shaw, 2006; Claringbould and Knoppers, 2008; Terjesen et al., 2009; Adriaanse and Schofield, 2013; Gaston et al., 2020) and less on (grassroots) sports clubs and volunteering. This is where our own study comes in. Based on a quantitative population survey on volunteering in Germany, we look for factors and interactions that can predict women's volunteering and leadership in sport. The following questions are guiding:

*RQ1*: What gender differences can be identified in access to volunteering in sport and in taking on leadership positions in Germany? What trends are emerging?

*RQ2*: Which variables predict (a) women's volunteering and (b) women's likelihood of taking on a leadership position and to what extent do differences with men emerge?

*RQ3*: What differences in (a) volunteering and (b) leadership positions can be identified in subgroups of women (as opposed to men)?

### Theoretical Background and Previous Empirical Findings

**Figure 1:**
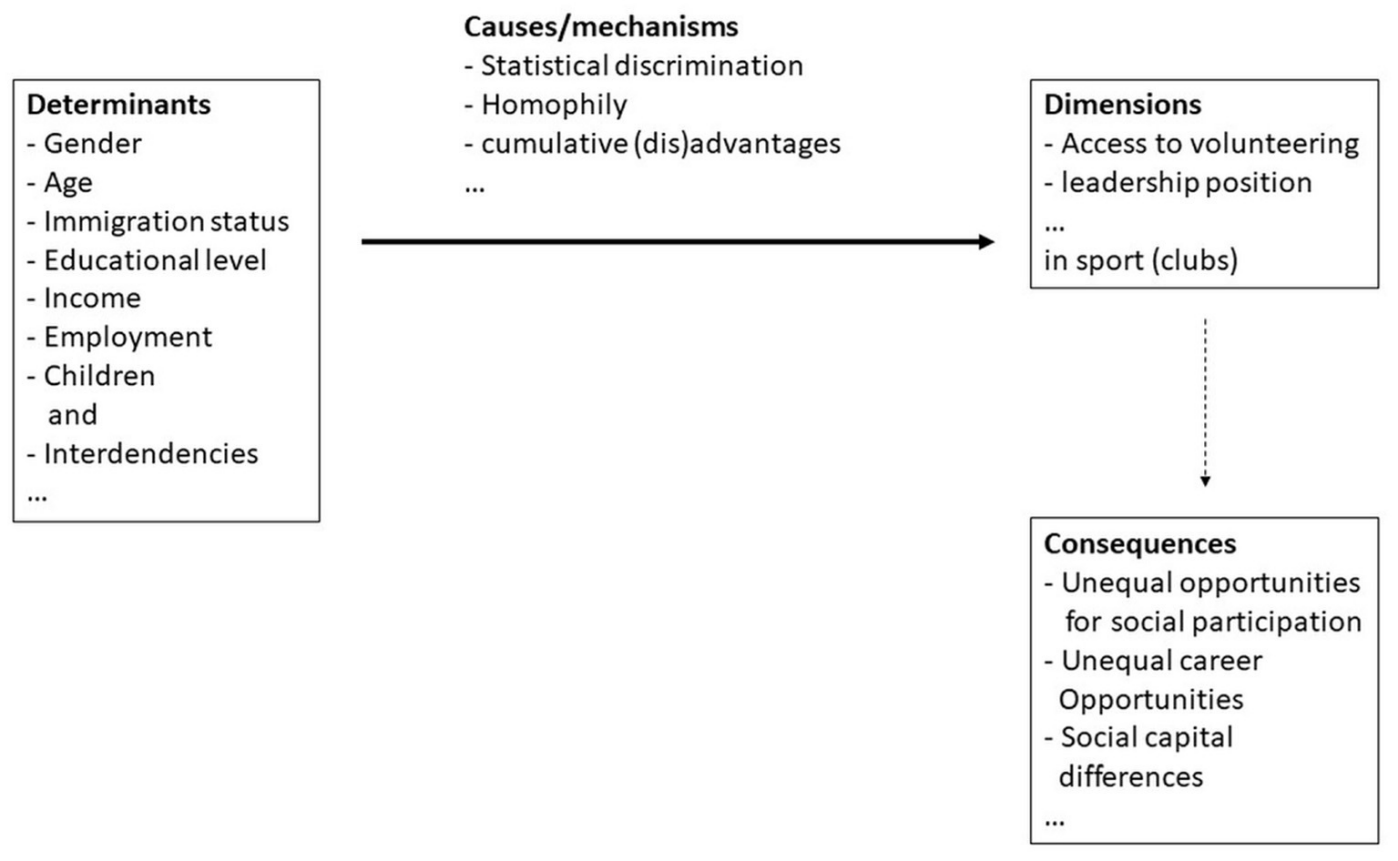
Volunteering as a dimension of social inequality (modified according to Solga et al., 2009, p. 17 and Rameder, 2015, p. 54).

In order to be able to analyze the unequal access to volunteering and leadership positions in sport, reference is made to inequality theory and intersectional approaches. The process model of Solga et al. (2009), with four structural levels of social inequality, can be used to theoretically discuss the unequal access to volunteering by women in sport and the resulting consequences (e.g., on opportunities for social and societal participation or on the generation of social capital). It provides a structuring scheme for the state of research and enables the disclosure of existing research gaps. On the input side, there are the so-called (1) determinants of social inequality, which lead to social inequality in different (2) dimensions through (3) mechanisms on the output side that need to be defined in more detail, which in turn are associated with certain (4) consequences (see Figure 1).

In our contribution, volunteering is considered to be a dimension of social inequality. At the same time, sports clubs as organizations provide a context for the genesis and reproduction of social inequality (Rameder, 2015).^1^ The unequal access to voluntary work for women and men and gender-specific forms of voluntary engagement are examples of horizontal gender segregation. Gender differences in leadership positions in sports clubs are an example of vertical gender segregation. The empirical finding that men are more likely to volunteer than women is initially only evidence of heterogeneity. Attention must also be directed to the consequences of unequal access in order to identify social inequality. Positive consequences of volunteering could be the generation of social capital (Putnam, 2000; Braun, 2007), the acquisition of skills (Heckman et al., 2006; Ruiter and De Graaf, 2009) or an increase in subjective wellbeing (Wicker and Downward, 2019).

Gender, class, immigration status, age and family status, among others, are conceived as determinants of social inequality. Belonging to these social groups is the basis for advantages and disadvantages in certain conditions of action and life (Solga et al., 2009). Gender differences in volunteering have already been reported. Class or access to various socio-economic and cultural resources such as paid employment, income and education are discussed as determinants of access to volunteering. There is relatively consistent evidence of a middle-class bias in volunteering (Rameder, 2015; Meusel, 2016; Simonson et al., 2022a). Moreover, previous findings suggest that immigrants are less likely to volunteer than non-immigrants (Rameder, 2015; Simonson et al., 2022a). Age differences in volunteering are also empirically documented. People between the ages of 30 and 49 are the most likely to volunteer (Simonson et al., 2022a).

Rameder (2015) suggests statistical disadvantage, the principle of homophily and the “Matthew effect” as examples of inequality-generating mechanisms. Statistical disadvantage occurs when decisions about individuals are made on the basis of behavioral assumptions regarding entire social groups (Solga et al., 2009). For example, association officials may believe that women are less flexible than men in terms of time due to household and childcare responsibilities. The principle of homophily can be defined as the tendency of people to seek out or be attracted to others who are similar to them. In this respect, contact between similar people occurs more frequently than between dissimilar people (McPherson et al., 2001). A distinction is also made between status homophily and values homophily. For example, it might be much more difficult for women of color to volunteer in a sports club that is strongly dominated by white men. The Matthew Effect or cumulative (dis)advantage is a general mechanism for inequality in any temporal process, such as in the life course, where a favorable relative position becomes a resource that generates further relative gains (DiPrete and Eirich, 2006). For example, men may accumulate more advantages than women. Flexible careers and part-time employment of women due to child-rearing and unpaid domestic care could lead to further disadvantages, e.g., in the pension system or also in filling voluntary positions. The causes or concrete mechanisms of social inequality must be specified and named in the respective empirical study (Solga et al., 2009).

#### Some Explanations for Gender Differences

The underrepresentation of women volunteers and especially in leadership positions in sport demonstrates that sport is a gendered institution and that all processes in sport operate within a hegemonic masculine norm (Burton, 2015). According to (Acker, 1999; Werkmann, 2021), gender differences are evident at the level of organization (e.g., less power and salary), at the symbolic level (e.g., male stereotypes as successful and strong leaders), at the level of interactions (e.g., reproductions of images of gender) and at the subject level (e.g., self-presentation as a gendered member of an organization). Even if gender differences are no longer systematized in all organizations or fields, they are still likely to be relevant depending on context and situation and in most cases yield disadvantages for women (Heintz et al., 1997; Wilz, 2010).

In order to explain the gender gap in volunteer work and in leadership positions, it is not uncommon to rely on explanations regarding the disadvantage of women in the labor market. Discrimination against women in different fields of activity and in different hierarchical professional and management positions can be seen, among other things, in the unequal pay for men and women even with the same qualifications and in the same profession (Blau and Kahn, 2017; Wicker et al., 2021). Whether and to what extent people are gainfully employed is not only an essential prerequisite for material prosperity, but at the same time an essential precondition for participation in social life as a whole (Erlinghagen et al., 2016).

Occupational segregation in the labor market is interpreted in particular as the result of gender socialization processes—e.g., being socialized into rigid gender roles—and as the result of structural constraints, obstacles and closure and integration processes located in the professional world (Werkmann, 2021). It is assumed that professional and non-professional spheres interact and that there is a relationship between paid and unpaid work. Rotolo and Wilson (2007) formulate two assumptions: The contrast hypothesis suggests that gender segregation in other spheres, such as gainful employment, is reversed or at least negated in voluntary work. Volunteering should provide women with an alternative role that could act as a substitute for paid careers. The spillover hypothesis assumes that gender segregation in volunteering mirrors or reproduces that in other work contexts. The empirical results tend to support the spillover hypothesis, including (or especially) in the field of sport (Neumayr and More-Hollerweger, 2009; Stadelmann-Steffen et al., 2010; Rameder, 2015; Braun, 2017). Rotolo and Wilson (2007) explain this finding with the fact that voluntary work is often organized in a similar way to paid work and domestic and family work, and that the tasks and positions in voluntary work are often just as hierarchically structured.

Meanwhile, similar mechanisms can be identified to explain the underrepresentation of women in leadership positions in sport organizations. First, gender-stereotypical assumptions in conjunction with expectations of leaders contribute to the fact that women are less likely to take on voluntary positions or even to be approached/recruited (Hoyt, 2005). Second, formal and informal structures of (sports) organizations (e.g., male networks, glass ceiling) create barriers to taking on volunteer leadership positions (McPherson et al., 2001). Third, gender-related problems and discrimination within organizations are not perceived by members; it is assumed that the sport system is gender-neutral. This is expressed, for example, in the statement that gender is not relevant in the recruitment of volunteers, but only the existing skills and qualifications (Combrink, 2004; Werkmann, 2021). Fourth, the question of reconciling family responsibilities is likely to be more important for women than for men, not only in terms of participation in gainful employment, but also in terms of participation in voluntary work, which is likely to be more time-consuming in management positions (Erlinghagen et al., 2016). Finally, negative stereotypes about women in sport and volunteering are also amplified by a lack of female representation in sport media, and specifically in traditionally male-dominated sports (Evans and Pfister, 2021). A number of (mostly qualitative) studies reconstruct these mechanisms. The reproduction of gender stereotypical norms and expectations occurs in sport organizations through language, culture and politics, as well as through other stakeholders such as sponsors, media or fans (in sum Evans and Pfister, 2021; see also Shaw and Slack, 2002; Claringbould and Knoppers, 2012; Schull et al., 2013). Several studies show that the uncritical acceptance of gendered roles and practices can result in gender stereotyping and in the construction of gendered identities and interactional politics (Hovden, 2010; Piggott and Pike, 2020).

#### The Need for Intersectional Analyses

As already explained, unequal access to volunteering cannot be explained one-dimensionally. “Differences among women thus become invisible, or at least overshadowed, by focusing on gendered binaries. The differences submerged within a single sex variable do not drown out all women equally either” (Harell, 2017, p. 498). The chance to hold a voluntary position in a sports club as an immigrant woman is probably lower than that of an immigrant man, but also than that of a woman without an immigration status. Therefore, intersectional analyses that focus on the interdependencies of social categories and inequalities are required (Acker, 2006; Melton and Bryant, 2017; Elling et al., 2019). However, intersectional analyses are rare in research on volunteering.

Volunteering can be seen mainly as a donation of working time. Whether and to what extent women and men can freely dispose of time is, among other factors, influenced by the amount of available economic capital (Bourdieu, 2005; Rameder, 2015). Women and men with high incomes can obtain more of their own time by using services, for example, while those with low incomes have to supplement their budgets with second jobs and are likely to have correspondingly less free time (including for voluntary work). In her qualitative study with socially disadvantaged people, Munsch (2005) points out that the fundamental areas of “family” and “gainful employment” must first be secured in order to engage in voluntary work. Assuming interactive rather than additive effects between gender and class, low economic resources are likely to play a different role for women with low economic capital than for men with low economic capital (Harell, 2017). Women work part-time more often than men, and they take care of children and relatives to a greater extent. This is likely to be at the expense of voluntary activity in sport. The negative effect of having little children is stronger for mothers than fathers volunteering. However, the effect is reversed for school-age children (Musick and Wilson, 2008). The latter could only be partially confirmed in Austrian studies (Neumayr and More-Hollerweger, 2009; Rameder, 2015).

In their analyses, Musick and Wilson (2008) could not find a moderating effect of gender on the relationship between educational level and volunteering in different areas. Recent studies from Germany have found that women with little educational attainment have the lowest rates of volunteering (Simonson and Hameister, 2017). Previous findings (Rotolo and Wilson, 2007) also indicate that the filling of leadership positions in many areas of voluntary work, including in sport, follows the implicit and explicit rules of the filling of positions in gainful employment. Rameder (2015) concludes that in the field of sport in Austria, men between 30 and 49 years of age with a high endowment of cultural capital, who are professionally active as managers or self-employed, are entrusted with voluntary leadership functions more often than average. In addition, people with an apprenticeship degree who are professionally successful also seem to make the leap into leading voluntary office, i.e., into functionary positions in sport. Administrative tasks correspond to middle-aged women with a medium endowment of cultural capital.

Harell (2017, p. 500) elaborates, “the gendered way in which immigration is experienced, as well as the ways in which resources (socioeconomic, linguistic capacity, social networks) are distributed among immigrant and non-immigrant populations, as well as within immigrant communities, should have profound effects on gender differences in participation among various communities”. In Canada, she found the largest gender differences in volunteering in sport, with men showing greater engagement rates than women in every ethnic group.

In summary, looking at volunteer careers, it can be said that female volunteers especially tended to have a higher level of education, were employed full-time and married. Most had children and had participated in sport since they were young (Cooper and Ayer, 2016; Melton and Bryant, 2017). In the study by Schlesinger et al. (2014), the lack of balance between school/occupation, family commitments and volunteering activities is the most frequent reason for constraints or for ending a volunteer career. It is likely that women especially report these compatibility problems (Cooper and Ayer, 2016; Baxter et al., 2021).

If we look at the career characteristics of volunteer managers in organized sport in Germany, they generally have a long sports club and competition biography and many years of club and official careers, regardless of gender. They feel closely connected to the club/association and on average have higher educational qualifications. Compared to male volunteer leaders, women are on average somewhat younger, less often married, less likely to have children, and less likely to conform to female stereotypes (summarized by Werkmann, 2021; see also Doll-Tepper and Pfister, 2004; Radtke, 2007, 2010).

Based on the state of research so far, we firstly assume that gender differences can also be observed in volunteering on a horizontal (access to voluntary work and tasks areas) and vertical level (holding a leadership position). Secondly, due to the gender and diversity strategies implemented in recent decades to recruit volunteers in sport, the longer-term trend is likely to show a narrowing of the gender gap in access to volunteering and the assumption of leadership positions. Thirdly, social structural determinants like gender, class or immigration status emerge as significant predictors of access to volunteering and leadership positions. Finally, it is assumed that socio-structural determinants are interwoven and that interaction effects between gender and other social features make an independent contribution to the variance explanation of the two dimensions of social inequality.

## Materials and Methods

### Dependent Variables—Dimensions of Social Inequality

Volunteering is defined as engagement that is (1) voluntary and (2) not aimed at material gain. The commitment (3) is oriented toward the common good, (4) takes place in the public sphere and (5) is usually carried out on a joint/cooperative basis (Enquete Commission, 2002). However, volunteering is also a specific form of work, since goods or services are produced in connection with an organization outside the household (e.g., in a sports club). While gainful employment is paid, voluntary work is unpaid. Volunteers may receive a small expense allowance (Meusel, 2016; Kelle et al., 2021).

#### Volunteering in Sport

In the 2014 and 2019 German Volunteer Surveys, all respondents were asked to indicate whether they had “participated somewhere (outside of work and family), for example, in an association, initiative, project, or self-help group” in the past 12 months (Kelle et al., 2021, p. 26). The question was intentionally broad. There was no restriction to a specific form of association, but a total of 14 different areas in which community activity can take place (e.g., “sports and exercise”, “leisure and socializing”, “culture and music”, “politics and political advocacy”) were listed. This also means that people who were active in sports did not necessarily have to be members of a sports club.

Only people who stated that they had been active in a field of activity in the last 12 months were asked—again with reference to the last 12 months—whether they also “perform voluntary work or are involved in associations, initiatives, projects or self-help groups” in these areas (Kelle et al., 2021, p. 28). Here, too, a broad understanding of engagement was deliberately applied, which, as it was also communicated to the respondents, “includes tasks and work (taken on) voluntarily, which one performs unpaid or for a small expense allowance” (Kelle et al., 2021, p. 28). A dummy variable “volunteering in sport” was coded with 1 = yes and 0 = no.

In order to be able to provide more detailed information on horizontal gender segregation in volunteering in sport, additional variables were included for descriptive evaluation.

#### Content of Volunteer Activities

Respondents were given nine items to describe their main activities: personal help, organizing/carrying out meetings and events, consulting, teaching assistance or guidance of a group, representation of interests or expressing opinions, public relations, administrative tasks, practical work which has to be done, fundraising. Each item could be answered with yes = 1 or no = 0.

#### Target Groups for Volunteering

For the question: Is your voluntary activity directed at a specific group of people? Nine different target groups were given: children and youth, families, older people, people with a handicap, people with an immigrant background, women, men, financially or socially disadvantaged people, people who need help or care. Each could be answered with yes = 1 or no = 0.

The dataset contains a question that can shed light on possible vertical gender segregation in volunteering.

#### Leadership Position in Voluntary Work

Men and women who spend the majority of their total volunteer time on sports-related activity were asked: “Do you have a management or board position?” (yes = 1 or no = 0).

### Independent Variables—Determinants of Social Inequality and Other Influencing Factors

The explanatory variables used in the analyses are the respective indicators for the ascribed features of gender, age and immigration status, as well as for the acquired features of level of education, employment, income and family status (Table 1).

**Table 1:** Independent variables—variable specification.

**Variables**	**Variable specification and coding**
**Determinants of social inequality**	
Gender	Female (0) Male (1)
Age	Age in years
Immigration status	No (0) Yes (1)
Employment	Full-time (1) Part-time/marginal (2) No (3)
Income	Household's monthly net income: Up to 3,000€ Over 3,000€
Education	No A-levels (0) A-levels (1)
Marital status	Single/unmarried (1) Married (2) Other (3)
Children under 18	Children over 18/no children in the household (0) Children under 18 in the household (1)
**Control variables I**	
Region	East-Germany (including Berlin) (0) West-Germany (1)
Years in the place of residence	Up to 10 years (0) More than 10 years (1)
Support from others	“If you need help, e.g., with errands, smaller jobs or looking after children or sick people: are there any people outside your household that you can turn to without paying them?” no (0) Yes (1)
Neighborhood cohesion	“How good is the social cohesion in your neighborhood?” Very bad=1 to very good=5
Health status	“How do you rate your current state of health?” very bad=1 to very good=5
Volunteering in other areas	No (0) Yes (1)
**Control variables II (additional predictors for leadership positions)**	
Motives: (a) influence society (b) get together with other people (c) gain prestige and influence (d) increase qualifications (e) earn a little extra money (f) have fun	“Please tell me if you fully agree, partially agree, partly, rather not agree or do not agree at all with the following statements about your voluntary work.” Don't agree at all =1 to Fully agree =5
Impulse: (a) personal initiative (b) leading persons (c) family, friends	(a) “Did the initiative come from you or were you asked if you wanted to take on the duties?” (b,c) “Where did the impetus come from for you to take on this activity? I will read you out a few possibilities. Please tell me in each case if the statement applies or not.” No (0) Yes (1)
Requirements and knowledge acquisition: (a) specific training necessary (b) special knowledge (c) social skills (d) personal skills	(a) “Is specific training or further training required to carry out the activity?” (b, c, d) “Have you, in the course of your activity, acquired the following skills or knowledge?” No (0) Yes (1)

*German Survey on Volunteering (FWS)*.

Given the complexity of volunteering in modern societies, the inequality theory approach is only one possible approach. Different theories should thus ideally be linked (e.g., Einolf and Chambré, 2011). We therefore integrate further context variables and personality features into our investigation, which have proven to be relevant in previous studies on volunteering in sports (Schlesinger and Nagel, 2013; Hallmann, 2015; Wicker, 2017) and on volunteering in general (Clary et al., 1998; Musick and Wilson, 2008; Hustinx et al., 2010). Region, years of residence, voluntary work in other areas, level of neighborhood cohesion, support from others and health status of respondents are included as control variables in all analyses. In order to predict leadership positions in sport, motives, information about impulse givers and necessary skills are additionally used as control variables (Table 1). The latter were only asked of volunteers.

### General Information on the German Survey on Volunteering and the Sample

At the end of the 1990s, the German government had a survey-based information system implemented that was intended to provide a representative empirical picture of volunteering in Germany over time. The goal of this system was to (further) develop a social strategy for promoting civil society on the basis of comprehensive data. A broad-based population survey was designed, which began in 1999 and has since been conducted five times. On the basis of the German Survey on Volunteering, it is possible to examine the scope and structure of voluntary engagement in the sports sector—in sports clubs as well as in self-organized projects and initiatives—on a cross-sectional basis, to map changes on a longitudinal basis and to discuss challenges faced by sports clubs with regard to human resources in the provision of services. The corresponding survey results have become an important tool for policy advice, especially in the field of “engagement policy”.

A large number of application-related problems played a relevant role in the conception of the German Survey on Volunteering and the construction of the corresponding survey instruments. Theoretical conceptualizations are therefore rather marginal. The process model of Solga et al. (2009) is intended to contribute in a pragmatic way to structuring and classifying the topics and variables of the German Volunteer Survey, to integrating existing findings and to guiding one's own empirical analyses. At this point, the limitations of the survey should be pointed out. For example, no information was collected on the respective organization (e.g., size of the club, sports offered, club goals and culture, socio-structural composition of the members). Nevertheless, the socio-structural characteristics of the respondents were asked in detail so that socio-structural analyses could be carried out.

Due to some fundamental methodological changes (Simonson et al., 2022b), our paper mainly analyses the results for volunteering in sport from the 2014 and 2019 waves. Older data are only used to describe long-term trends toward gender equality. The datasets are considered representative of the population of the Federal Republic of Germany regarding the characteristics of age from 14 years, gender, federal state and municipality size, class and education. The sample allows analyzing individual fields of action (e.g., the area of sport) and individual population groups (e.g., women and men).

In 2014 and 2019, computer-assisted telephone interviews (CATI) took place both via landline and mobile phone. In the landline interviews, the target person was selected from the household using the last-birthday method. In the cell phone interviews, the person who primarily uses the phone was interviewed (Simonson et al., 2017, 2022c). In 2014, the proportion of people contacted (*N* = 148,668) who took part in the interview was 21.3% (*N* = 31,600) or 19.3% (*N* = 28,690) based on the fully realized and analyzable interviews (Simonson et al., 2017). In 2019, 21.1% (*N* = 31,454) took part and 18.6% (*N* = 27,762) of 149,053 contacts resulted in analyzable data (Schiel et al., 2020). The response rate is similarly low as in other telephone surveys (Engel et al., 2012). In both waves, the main reasons for drop-out were refusal to participate on principle, lack of interest in the topic of the study or interviews in general and the length of the interview (Schiel et al., 2020). Distorting factors in the composition of the sample (e.g., a disproportionate sampling according to region) and unequal participation probabilities (e.g., according to age) were compensated by weighting. Weighting was based on the characteristics of federal state, community size class depending on the number of inhabitants and the population and job density (Behrens et al., 2019), gender, age group and school education (Simonson et al., 2022c). Data collection, data checking and weighting were the responsibility of the Institute for Applied Social Science (infas). The empirical analyses are based on the adult sample (18 years and older) because the study examines the roles of employment and the presence of children in the household as possible determinants of social inequality. Due to the age-related case selection, the sample size differs between the unweighted and the weighted sample (Table 2).

**Table 2:** Description of the overall sample.

	**2014**		**2019**		**2014 + 2019**
	**Unweighted**	**Weighted**	**Unweighted**	**Weighted**	**Weighted**
*N*	27,425	27,350	27,119	26,569	53,919
**Gender**					
Women	54.8	51.4	52.6	51.0	51.2
Men	45.2	48.6	47.4	49.0	48.8
Age	52.34 (17.26)	50.34 (18.43)	56.57 (16.72)	50.85 (18.70)	50.59 (18.56)
**Education**					
No A-levels	55.5	67.6	49.3	60.9	64.3
A-levels	44.5	32.4	50.7	39.1	35.7
**Region**					
West-Germany	65.5	79.9	66.6	80.3	80.1
East-Germany (including Berlin)	34.5	20.1	33.4	19.7	19.9

*Share of respondents in % or means and standard deviations. German Survey on Volunteering (FWS)*.

The following analyses focus on the one hand on the overall sample (Table 2) and on the other hand on the subsample sport (Table 3). The subsample consists firstly of the women and men who volunteer in the field of sport and physical activity. Secondly, we will look at the subgroup that spends the majority of their total volunteer time on sports-related activity and holds leadership positions there.

**Table 3:** Description of the subsample sport (weighted).

	**Volunteers in sport**		**Volunteering in sport as the most time-consuming voluntary activity**			
			**Volunteers**		**Leadership position**	
	2014	2019	2014	2019	2014	2019
*N*	3,910	3,521	2,832	2,455	826	669
**Gender**						
Women	40.4	44.4	37.0	41.6	29.1	32.5
Men	59.6	55.6	63.0	58.4	70.9	67.5
Age	46.12 (16.72)	46.54 (16.52)	45.65 (16.85)	46.60 (16.41)	48.90 (16.43)	49.43 (16.63)
**Education**						
No A-levels	56.4	48.4	58.7	49.5	62.1	51.9
A-levels	43.6	51.6	41.3	50.5	37.9	48.1
**Region**						
West-Germany	83.8	83.5	83.3	83.4	85.5	85.0
East-Germany (including Berlin)	16.2	16.5	16.7	16.6	14.5	15.0

*Share of respondents in % or means and standard deviations. German Survey on Volunteering (FWS)*.

### Analysis Strategies

The data were processed and analyzed in the statistical software IBM SPSS 25.0. In a first step, descriptive characteristics are calculated and statistical tests are conducted to identify gender differences. The analysis was carried out depending on the measurement level of the variables, in the case of categorical variables using contingency tables (cross-tabulations) and Chi^2^-Tests or in the case of metric variables *t*-tests. The longer trends (1999–2019) in terms of changes in the proportions of women and men volunteering or taking up leadership positions in sport (RQ1) are only presented descriptively due to the reported methodological problems. The changes between 2014 and 2019 are examined in the regression analyses.

To be able to answer the second question on the prediction of access to volunteering in sport and taking up leadership positions, binary-logistic regression models are calculated with the determinants of social inequality as predictors, controlling for contextual and personality features. To examine how gender and other variables jointly affect volunteering and the distribution of leadership positions, interaction effects were included. Appropriate prerequisites were previously tested and multicollinearity analyses were conducted. Ignoring the interactions, all of the correlations between the predictors are less than the threshold of r = 0.80, which is often used in regression analysis (Urban and Mayerl, 2018).

## Results

### Gender Differences in Volunteering in the Field of Sport

In 2019, 11.5% of women and 15% of men volunteered in sports. Women and men volunteer primarily because it is fun, they want to get together with other people, they want to help other people and influence society. Power and influence, as well as earning extra money, play a much smaller role. Women differ from men in their respective approval ratings. They agree more strongly with the former reasons, while they agree with the latter reasons less often than men (Table 4). Differences between survey dates are marginal.

**Table 4:** Reasons to volunteer, differentiated by gender and survey period.

**Motive** ** (1 = disagree-5 = agree)**	**2014**				**2019**			
	**Women**	**Men**	***t*** **(*****p*****)**	**d**	**Women**	**Men**	***t*** **(*****p*****)**	**d**
Influence society	4.34 (0.91)	4.24 (0.98)	−3.41^***^	0.11	4.34 (0.93)	4.14 (1.03)	−6.22^***^	0.20
Get together with other people	4.54 (0.78)	4.36 (0.87)	−6.97^***^	0.23	4.28 (0.93)	4.16 (0.98)	−3.58^***^	0.13
Gain prestige and influence	2.89 (1.26)	2.97 (1.27)	1.84		2.55 (1.22)	2.76 (1.22)	5.01^***^	0.17
Increase qualifications	3.29 (1.46)	3.38 (1.50)	1.89		3.47 (1.39)	3.43 (1.41)	−0.91	
Earn a little extra money	1.58 (0.94)	1.63 (1.04)	1.68		1.45 (0.85)	1.55 (0.94)	3.23^**^	0.11
Have fun	4.78 (0.54)	4.73 (0.61)	−2.82^**^	0.09	4.77 (0.56)	4.71 (0.61)	−3.16^**^	0.10
Do something for the common good	–	–	–	–	4.54 (0.76)	4.41 (0.81)	−4.79^***^	0.17
Give something back	–	–	–	–	3.90 (1.20)	3.90 (1.19)	0.18	
Help other people	–	–	–	–	4.55 (0.77)	4.47 (0.81)	−2.71^**^	0.10

*Gender differences using t-test:*

* *p < 0.05*,

** *p < 0.01*,

*** *p < 0.001; means, standard deviations, t-tests for independent samples and effect size Cohen's d. German Survey on Volunteering (FWS)*.

**Figure 2:**
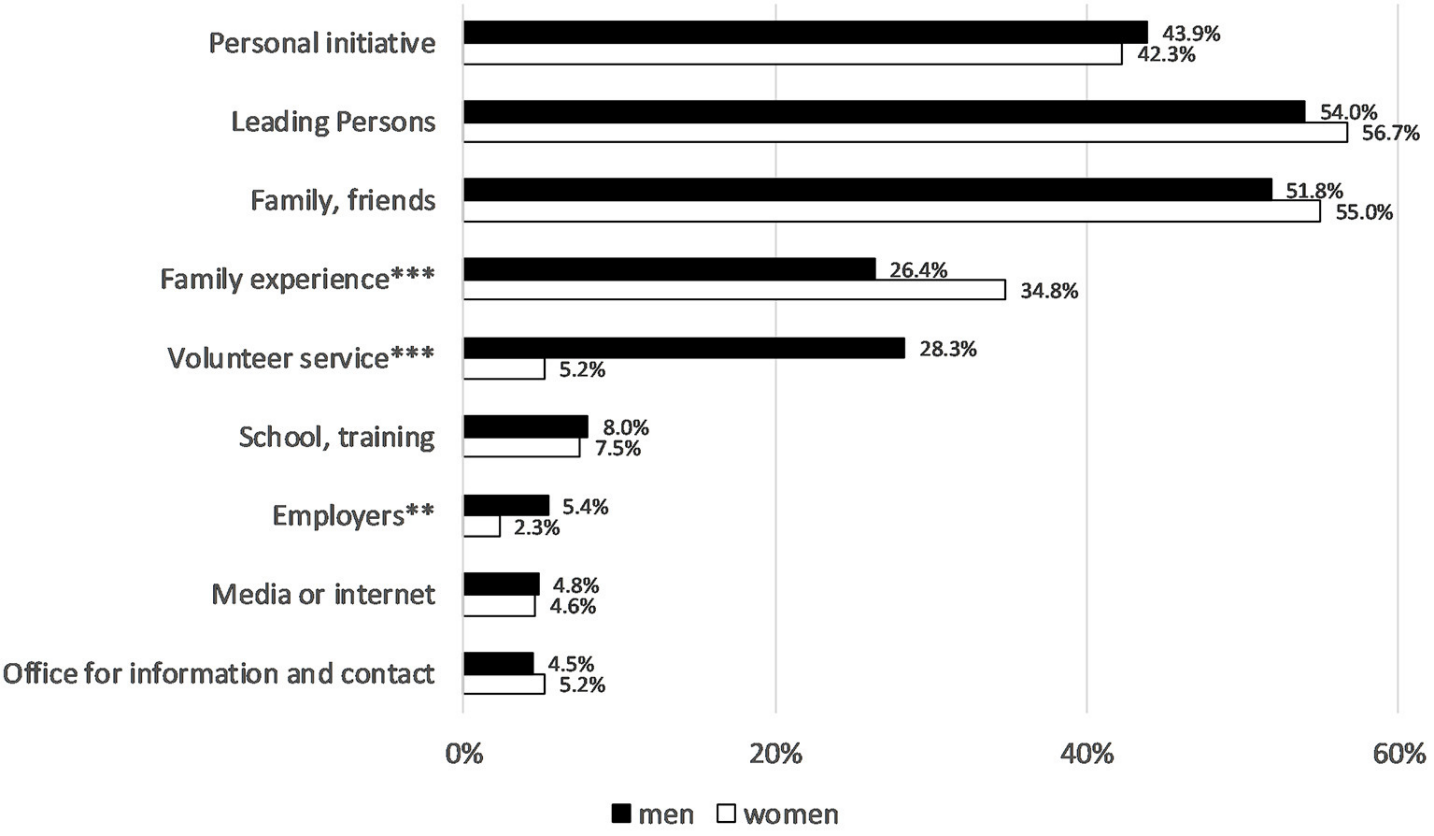
Impulses for volunteer activity in 2019. Approval ratings in %, gender differences using Chi^2^ test: *p < 0.05, **p < 0.01, ***p < 0.001. German Survey on Volunteering (FWS).

In about 43% of cases, the initiative to take on the voluntary work came from the volunteers themselves (women: 42.3%; men: 43.9%). Just over half of the respondents indicate that the impetus came from leaders within the club or group and in just as many cases the impulse came from family members, friends or acquaintances who were already active there. Family experiences—and as a tendency this applies also to family members, friends or acquaintances—are indicated more often from women as impetus, whereas voluntary service and the employer were more important for men (Figure 2).

**Figure 3:**
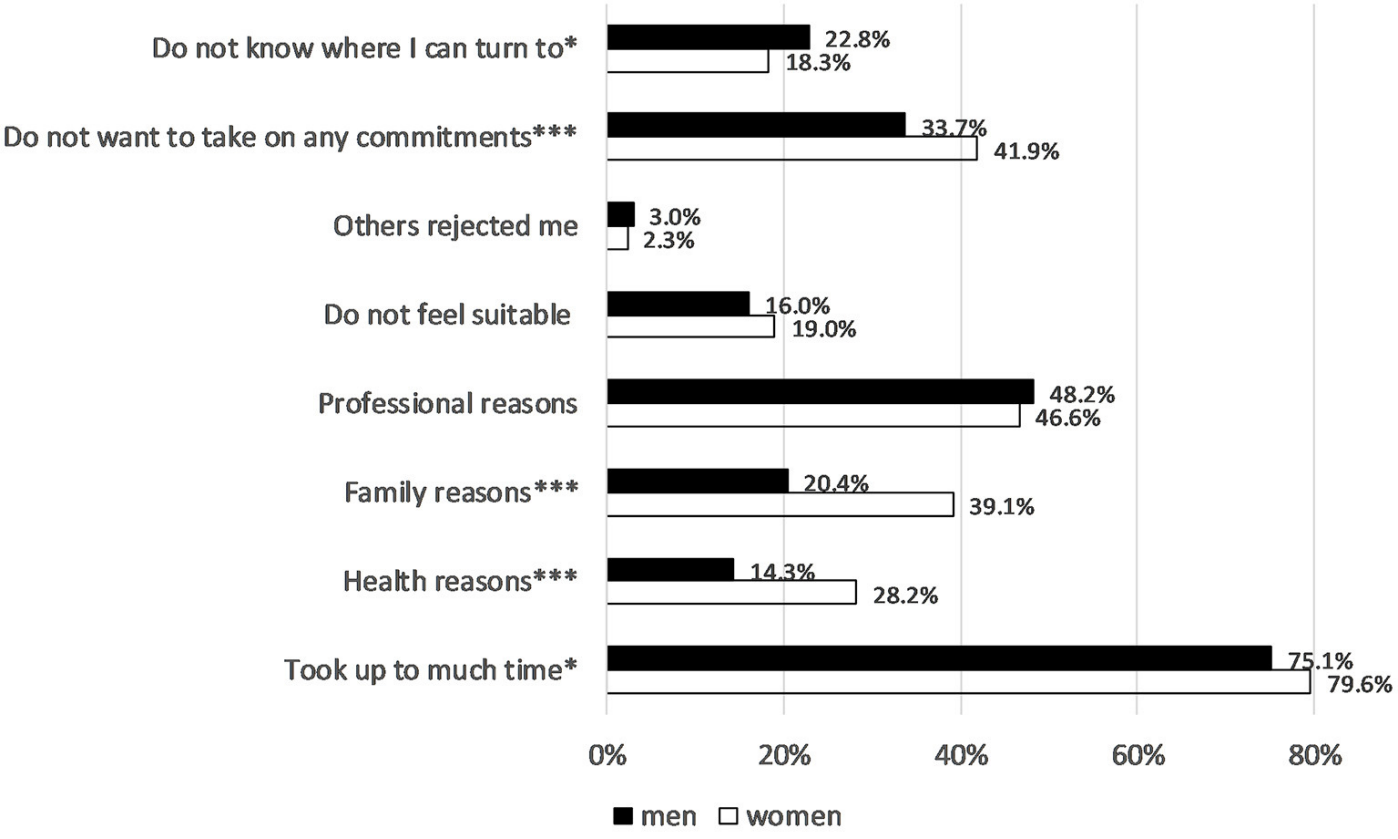
Reasons for not volunteering in 2019. Approval ratings in %, gender differences using Chi^2^ test: *p < 0.05, **p < 0.01, ***p < 0.001. German Survey on Volunteering (FWS).

From a comparative gender perspective, the reasons people who are active in sports do not take on voluntary work are also interesting. Most respondents overall, and women a little more often than men, consider time reasons to be decisive, while professional reasons are frequently cited by both sexes. Moreover, women mention family reasons and health reasons more often than men and state more often that they do not want to take on any commitments (Figure 3).

**Figure 4:**
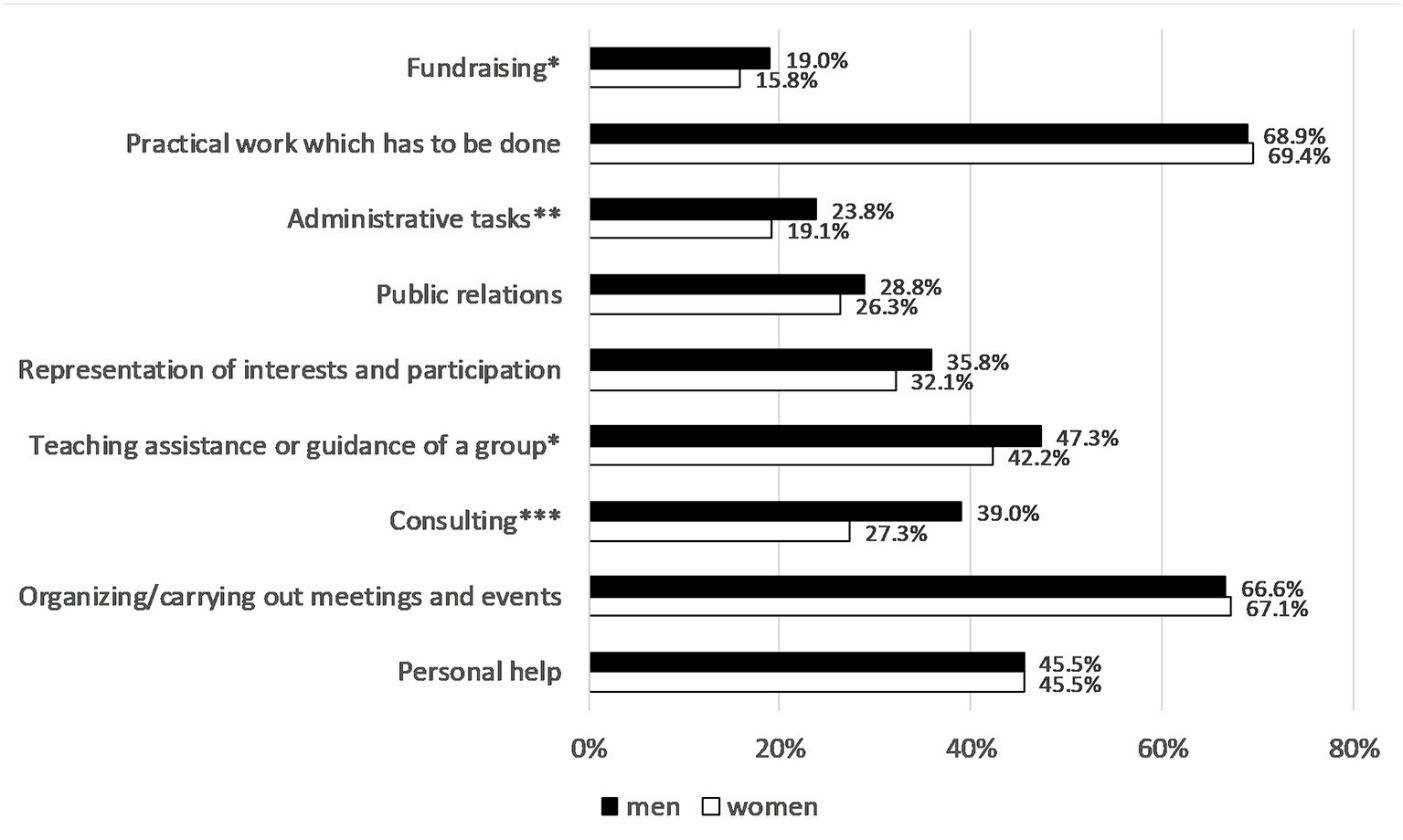
Content of volunteer activities in 2019. Approval ratings in %, gender differences using Chi^2^ test: *p < 0.05, **p < 0.01, ***p < 0.001. German Survey on Volunteering (FWS).

With regard to the volunteers with the most time-consuming voluntary activity in sports, there are more similarities than differences between women and men in the content of their activities (Figure 4). Practical activities, the organization and implementation of (sporting) events and personal help are mentioned particularly frequently. Men state more frequently than women that they take care of fundraising and administrative activities and that they perform consulting services.

In 2019, women named families significantly more often than men as a special target group for their volunteer work. In 2014, this applied to families and also to older people, people with a handicap and people who need help or care. In 2014 and 2019, women were more involved with women and men more with men (Table 5).

**Table 5:** Target groups for volunteering, differentiated by gender and survey period.

	**2014 (*****N*** **=** **2,831)**				**2019 (*****N*** **=** **2,453)**			
**Target groups**	**Women**	**Men**	**X** ^**2**^	**CC**	**Women**	**Men**	**X** ^**2**^	CC
Children and youth	59.0	59.0	0.0		59.0	60.3	0.4	
Families	32.1	23.6	24.3^***^	0.09	34.0	27.2	13.1^***^	0.07
Older people	26.4	21.0	11.3^***^	0.06	24.2	24.2	0.0	
People with a handicap	8.5	4.7	17.0^***^	0.08	10.4	9.0	1.3	
People with an immigrant background	9.1	8.9	0.0	0.00	9.3	12.5	6.2^*^	0.05
Women	21.8	7.5	120.0^***^	0.20	21	8.0	91.4^***^	0.19
Men	7.5	31.1	210.6^***^	0.26	6.4	28.0	180.6^***^	0.26
Financially or socially disadvantaged people	7.2	7.6	0.1		7.9	10.4	4.4^*^	0.04
People who need help or care	5.3	3.2	7.7^**^	0.05	5.6	7.6	3.8	

*Gender differences using Chi^2^ test:*

* *p < 0.05*,

** *p < 0.01*,

*** *p < 0.001; CC: Contingency Coefficient. Frequencies in %. German Survey on Volunteering (FWS)*.

In 2014 and 2019, 84.1% of the women and 87.6% of the men with the most time-consuming voluntary activity in sports are involved in (formal) sports clubs. Women spend an average of 3.0 h and men more than 4.2 h per week on their most time-consuming voluntary activity. Men have been doing this for an average of 12.1 years, women for 10.2 years.

### Trend Analyses on the Gender Gap in Volunteering and in the Holding of Leadership Positions in Sport

**Figure 5:**
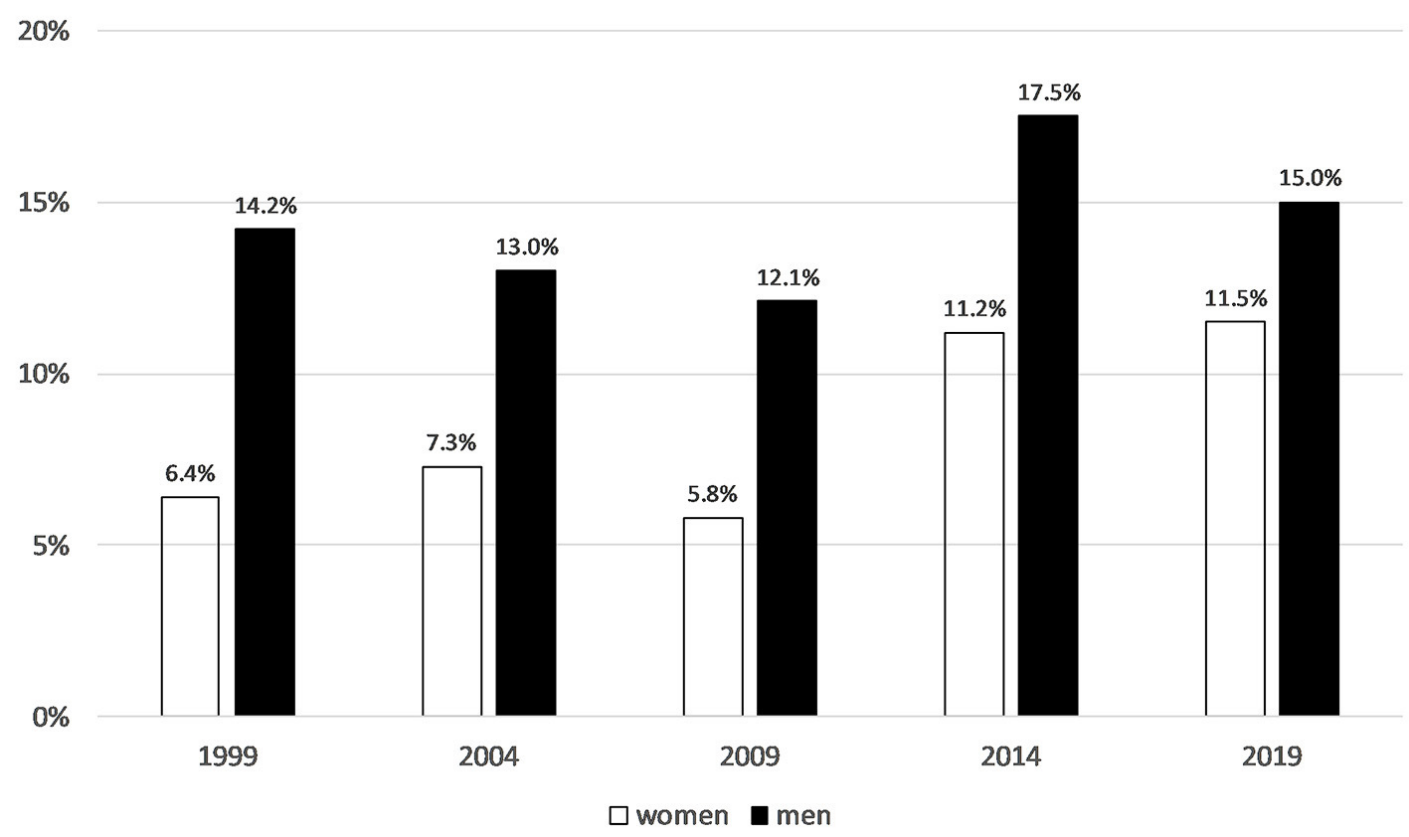
Population share of people volunteering in sport and physical activity (1999 *N* = 14,155, 2004 *N* = 14,217, 2009 *N* = 19,048, 2014 *N* = 27,350, 2019 *N* = 26,568). German Survey on Volunteering (FWS).

Trend analyses of the German Survey on Volunteering show that women have caught up with men in terms of the proportion of people volunteering. Whereas, in 1999 6.4% of women were volunteering in sport, 20 years later the figure was 11.5%. The proportion of volunteers among men was 14.2% in 1999 and 15.0% in 2019, with major fluctuations in between (Figure 5). Some methodological changes were made to the Survey in 2014, so trends in volunteering before and since 2014 should be considered separately. However, the reduction in gender differences in 2019 is due to the lower proportion of engaged men.

**Figure 6:**
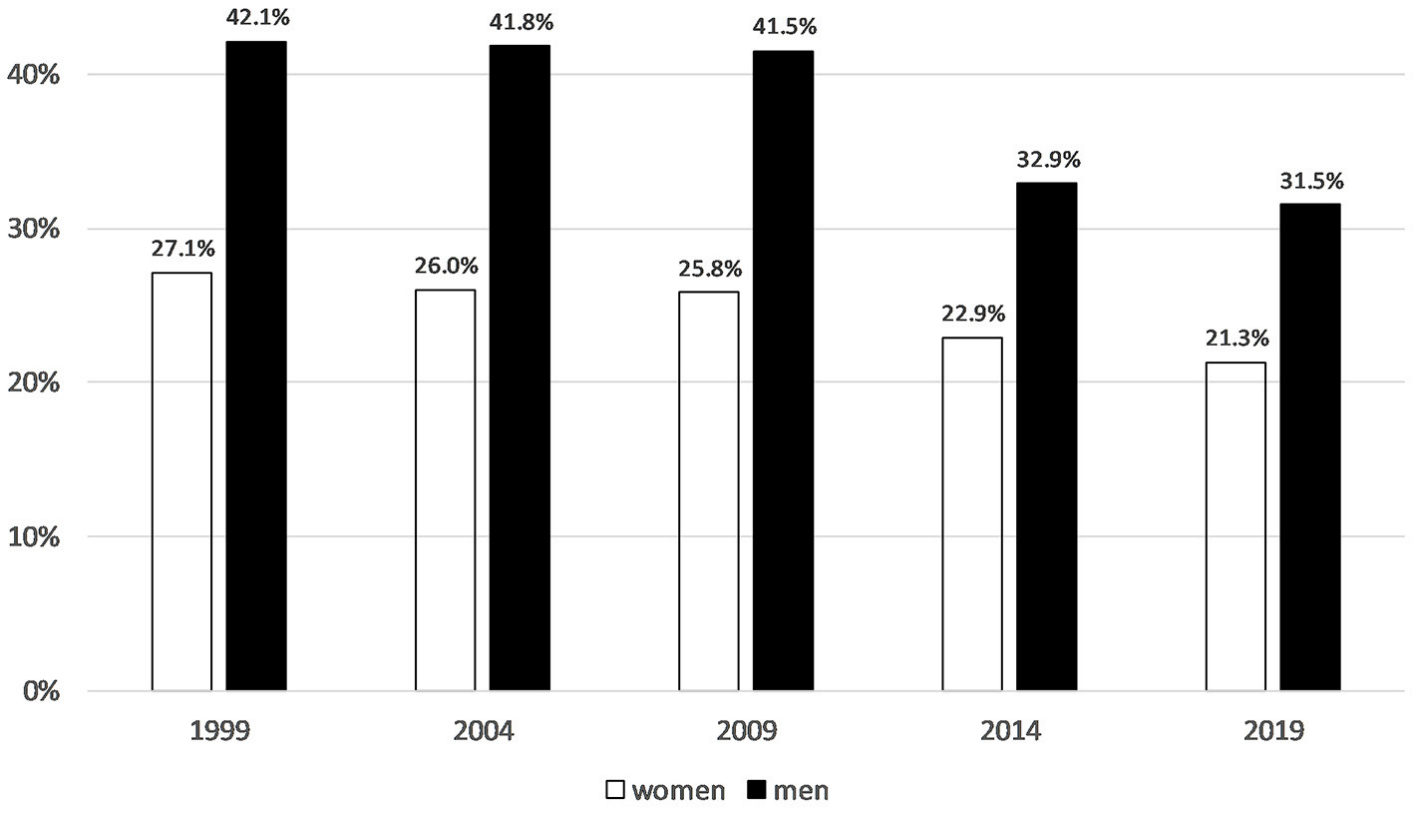
Proportion of persons in volunteer leadership positions among persons with the most time-consuming voluntary activity in the sports sector (1999 *N* = 1,192, 2004 *N* = 1,166, 2009 *N* = 1,336, 2014 *N* = 2,832, 2019 *N* = 2,454). German Survey on Volunteering (FWS).

Women continue to be underrepresented in volunteer leadership positions in sport. The gender gap was around 15 percentage points before 2014 and around 10 percentage points since 2014, to the disadvantage of women in leadership positions (Figure 6).

### Predictors of Voluntary Engagement or Leadership Positions in Sports

Table 6 shows the results of the regression analyses examining the predictors of volunteering. Almost all in Model 1 included variables—operationalized as determinants of social inequality—contribute significantly to the prediction of access to volunteering. Model 1 contains only the main effects. The proportion of explained variance is rather low at 14.2%.

**Table 6:** Predictors of volunteering in sport in 2014 and 2019.

	**Model 1**			**Model 2**		
	**B**	**SE**	**Odds ratio**	**B**	**SE**	**Odds ratio**
**Main effects**						
Gender: Women	−0.42	0.03	0.65^***^	−0.54	0.25	0.58^*^
Employment (Ref. full-time)						
part-time/marginal	0.10	0.05	1.10^*^	−0.28	0.11	0.76^*^
no	−0.15	0.04	0.86^***^	−0.05	0.05	0.95
Income (>3000€)	0.37	0.03	1.45^***^	0.44	0.04	1.55^***^
Education: A-levels	0.20	0.03	1.22^***^	0.17	0.04	1.18^***^
Immigration status (yes)	−0.74	0.04	0.48^***^	−0.74	0.06	0.48^***^
Age	−0.02	0.00	0.98^***^	−0.02	0.00	0.98^***^
**Marital status (Ref. single/unmarried)**						
married	0.21	0.04	1.23^***^	0.26	0.06	1.29^***^
other	−0.02	0.06	0.98	−0.05	0.09	0.95
Children under 18 (yes)	0.03	0.04	10.03	0.01	0.05	1.01
Region: East-Germany	−0.27	0.04	0.77^***^	−0.26	0.05	0.77^***^
Years in the place of residence (>10 years)	0.39	0.04	1.48^***^	0.41	0.05	1.51^***^
Neighborhood cohesion (1 = low – 5 = high)	0.15	0.02	1.17^***^	0.14	0.02	1.16^***^
Support from others (yes)	0.32	0.05	1.38^***^	0.31	0.06	1.37^***^
Health status (1 = bad – 5 = good)	0.14	0.02	1.15^***^	0.14	0.02	1.15^***^
Volunteering in other areas (yes)	0.81	0.03	2.24^***^	0.70	0.04	2.01^***^
Time (2019)	−0.15	0.03	0.86^***^	−0.19	0.04	0.83^***^
**Interaction Effects** ^a^						
Gender × Employment (Ref. full-time)						
Gender x part-time/marginal				0.41	0.12	1.50^**^
Gender x no				−0.23	0.07	0.79^**^
Gender × Income (>3,000€)				−0.17	0.07	0.84^**^
Gender × Volunteering in other areas (yes)				0.26	0.06	10.30^***^
Nagelkerke R^2^	0.142			0.145		
*N* (weighted)	42,720			42,720		

*+p < 0.1*,

* *p < 0.05*,

** *p < 0.01*,

*** *p < 0.001*.

*a Only significant interaction effects are reported*.

*Binary logistic regression*.

*German Survey on Volunteering (FWS)*.

To examine how gender and other variables jointly affect volunteering, interaction effects between the gender variable and the other variables were included in Model 2. The results show that part-time and marginal employment are more likely to be associated with volunteering among women than among men (Tables 6, 7). However, the likelihood of volunteering decreases more for women than for men when they are not employed at all. Moreover, higher income for women is less likely to be associated with voluntary work than it is for men, while volunteering in other areas is more likely to have positive effect on volunteering in sports for women than for men. Compared to Model 1 the proportion of explained variance increases only slightly to 14.5% by adding interaction effects. The odds ratios for the significant interaction effects are rather small.

**Table 7:** Proportion of volunteers by gender and different moderator variables.

**Moderator variable**	**Value**	**Proportion of women volunteering**	**Proportion of men volunteering**
Employment	Part-time/marginal	18.1 (893)	13.0 (115)
	No	7.8 (891)	13.2 (1,177)
	Full-time	14.1 (726)	19.8 (2,265)
Income	>3,000 Euro	18.5 (1,187)	24.4 (1,942)
	≤ 3,000 Euro	8.8 (1,323)	12.1 (1,615)
Volunteering in other areas	Yes	20.7 (1,558)	26.1 (1,925)
	No	6.8 (952)	11.8 (1,633)

*Proportions in % (N). German Survey on Volunteering (FWS)*.

In order to find out which variables (in addition to socio-structural characteristics) predict a volunteer leadership position in sports, motives, requirements and impetus for volunteering are used, among others (Table 8). For these analyses, only those who perform their most time-consuming voluntary activity in sports are included. Model 1 contains only the main effects. The proportion of explained variance is rather low at 13.6%.

**Table 8:** Predictors of leadership positions in sport in 2014 and 2019.

	**Model 1**			**Model 2**		
	**B**	**SE**	**Odds ratio**	**B**	**SE**	**Odds ratio**
**Main effects**						
Gender (Women)	−0.45	0.09	0.64^***^	0.20	1.05	1.22
Employment (Ref. full-time)						
Part-time/marginal	0.00	0.13	1.00	0.64	0.27	1.90^*^
No	0.05	0.09	1.06	0.08	0.11	1.09
Income (>3,000€)	0.22	0.08	1.24^**^	0.14	0.10	1.15
Education (A-levels)	−0.12	0.08	0.88	−0.08	0.10	0.92
Immigration background (yes)	−0.25	0.11	0.78^*^	−0.30	0.14	0.74^*^
Age	0.01	0.00	1.01+	0.01	0.00	1.01
Marital status (Ref. single/unmarried)						
Married	0.25	0.12	1.28^*^	0.35	0.15	1.42^*^
Other	0.29	0.16	1.34+	0.39	0.21	1.47+
Children under 18 (yes)	−0.30	0.10	0.74^**^	−0.45	0.12	0.63^***^
Region: East-Germany	−0.13	0.10	0.88	−0.02	0.12	0.98
Years in the place of residence (>10 years)	0.23	0.09	1.26^*^	0.31	0.11	1.36^**^
Neighborhood cohesion (1 = low – 5 = high)	0.02	0.04	1.02	0.03	0.05	1.03
Support from others (yes)	0.23	0.12	1.26+	0.29	0.14	1.34^*^
Health status (1 = bad – 5 = good)	0.02	0.04	1.02	0.04	0.06	1.04
Impulse: yes						
Personal initiative	−0.30	0.08	0.74^***^	−0.25	0.10	0.78^*^
Leading persons	0.46	0.08	1.58^***^	0.41	0.10	1.51^***^
Family, friends	−0.35	0.07	0.70^***^	−0.22	0.09	0.80^*^
Motive (1 = disagree – 5 = agree)						
Influence society	0.14	0.04	1.15^***^	0.16	0.05	1.17^***^
Get together with other people	0.15	0.05	1.16^***^	0.16	0.05	1.17^**^
Gain prestige and influence	0.03	0.03	1.03	0.03	0.04	1.03
Increase qualifications	0.02	0.03	1.02	0.01	0.03	1.01
Earn a little extra money	−0.14	0.04	0.87^**^	−0.16	0.05	0.85^**^
Have fun	−0.10	0.06	0.90	−0.10	0.08	0.90
Requirements and knowledge acquisition (yes)						
Specific training necessary	0.06	0.09	1.06	−0.12	0.11	0.89
Specific knowledge	0.64	0.08	1.90^***^	0.56	0.10	1.74^***^
Social skills	−0.04	0.09	0.96	−0.15	0.11	0.86
Personal skills	0.23	0.08	1.25^**^	0.23	0.10	1.26^*^
Time (2019)	−0.05	0.08	0.95	−0.02	0.09	0.98
**Interaction effects** ^**a**^						
Gender × Employment (Ref. full-time)						
Gender × part-time/marginal				−0.98	0.32	0.38^**^
Gender × children under 18 (yes)				0.49	0.21	1.63^*^
Gender × impulse (yes)						
Gender × family, friends				−0.37	0.16	0.69^*^
Gender × requirements and knowledge acquisition (yes)						
Gender × specific training necessary				0.47	0.19	10.60^*^
Gender × social skills				0.36	0.20	1.43+
Nagelkerke R^2^	0.136			0.154		
*N* (weighted)	4,135			4,135		

*+p < 0.1*,

* *p < 0.05*,

** *p < 0.01*,

*** *p < 0.001*.

a *Only significant interaction effects are reported*.

*Binary logistic regression*.

*German Survey on Volunteering (FWS)*.

Again, Model 2 contains interaction effects. When predicting leadership positions, considering interaction effects has a greater impact than when predicting volunteering. The increase of explained variance to 15.4% and the odds ratios for the significant interaction effects are higher. This suggests that gender differences as a function of other variables are larger for leadership positions than for volunteering. Here, the scope of employment seems to be of particular importance. Part-time and marginal employment increases the likelihood of having a leadership position to a greater extent for men than for women whereas it is the other way around for volunteering. In the context of leadership positions, men benefit more than women if there are no children in the household. Furthermore, women seem to be moving more into leadership positions, if they have the impression that specific training is necessary for voluntary work and that social skills can be acquired through voluntary work. Getting the impetus to do voluntary work from family or friends worsens the odds of occupying a leadership position more significantly for women than for men (Tables 8, 9).

**Table 9:** Proportion of volunteers in leadership positions by gender and different moderator variables.

**Moderator variable**	**Value**	**Proportion of women in leadership positions**	**Proportion of men in leadership positions**
Employment	Part-time/marginal	22.2 (119)	39.1 (27)
	Full-time	24.2 (122)	30.9 (527)
Children under 18	Yes	22.2 (121)	26.9 (197)
	No	23.5 (240)	35.5 (651)
Impulse: family, friends	Yes	17.8 (153)	29.9 (376)
	No	29.3 (208)	36.0 (472)
Requirements and knowledge: specific training necessary	Yes	34.7 (129)	34.5 (228)
	No	19.4 (232)	32.5 (619)
Requirements and knowledge: social skills	Yes	25.3 (277)	32.8 (620)
	No	17.8 (84)	33.5 (228)

*Proportions in % (N)*.

*German Survey on Volunteering (FWS)*.

## Discussion

First of all, gender differences still exist both at a horizontal level (access to voluntary activities in sport, target groups and contents of these voluntary activities) and on a vertical level (assumption of leadership positions). The proportion of women who volunteer and who take on leadership positions is lower than the proportion of men. In 2014, gender-stereotyped tasks and responsibilities were still evident. Gender differences are also evident in the impulses and motives for taking up volunteer work in sport. Although men and women consider the same motives important (social reasons, common good, enjoyment of the activity) or unimportant (power and influence, earning money), their level of agreement differs. The situation is similar when it comes to impulses. However, family and friends as well as family experiences are mentioned more often by women, while voluntary service as well as the employer are mentioned more often by men as impulses. Nevertheless, every second woman and every second man are recruited for a volunteer position by older members of the club.

The gender differences were less pronounced in 2019. In the long-term trend, too, the gender gap seems to be narrowing somewhat, at least as far as access to voluntary work in sport is concerned. However, this trend is not yet apparent when it comes to taking on leadership positions in sport clubs. Moreover, the narrowing of the gender gap can rather be attributed to the fact that the proportion of men volunteering is declining. The proportion of female volunteers, however, has barely increased.

In the context of social inequality, ascribed characteristics such as gender, age and immigration status, as well as acquired characteristics such as education level, employment and income are of central importance for the reproduction of unequal living conditions of women and men. This applies in particular to access to volunteering. The determinants identified for gainful employment and also mechanisms for the (re)production of social inequality also seem to be at work in access to volunteering in sport. Once access to volunteering in sport has been mastered, socio-structural characteristics seem to have less explanatory power than contextual and personality characteristics in predicting leadership positions in sport. But even in the already selective sub-sample of volunteers in sport, indications of classic patterns of hierarchization in the occupation of positions can be found. A high income increases and an immigration background decreases the probability of taking on leadership positions in sport. However, motives, perceived requirements and the source of inspiration, among other factors, also differ according to gender, so that social inequalities can also be reproduced via these features.

Recent studies point to the relevance of interdependencies between gender and other socio-structural characteristics in explaining unequal access and unequal positions in voluntary work (e.g., Musick and Wilson, 2008; Rameder, 2015). Gender segregation in occupational work is also indirectly reflected in the findings on voluntary engagement. Women work disproportionately part-time. They are also more likely than men to cite family and time-related reasons (e.g., lack of compatibility between family, work and volunteering) for not volunteering. Disordinate interactions are evident in access to volunteering between gender and employment. As expected, part-time and marginal employment is more often associated with volunteering among women than among men (Rameder, 2015). When predicting leadership positions, it is the other way around. Part-time and marginal employment increase the likelihood of having a leadership position to a greater extent for men than for women. Very few men are part-time or marginally employed and presumably, women in part-time and marginal employment are often responsible for unpaid domestic and family work (Destatsis, 2022). As a result, women are not only less likely to take up management positions in professional life, but are also more likely to be prevented from expanding their voluntary work by taking on a more time-consuming leadership position. Gender differences as a function of other variables are larger for leadership positions than for volunteering which is partly due to additional predictors for leadership positions and their interactions with gender. Men benefit more than women if there are no children in the household. Women seem to be moving more into leadership positions, if they have the impression that specific training is necessary for voluntary work and that social skills can be acquired through voluntary work. Getting the impetus to do voluntary work from family or friends worsens the odds of occupying a leadership position more for women than for men.

The present study has some limitations that may guide future research. Due to methodological changes, the survey waves of the German Volunteer Survey from 2014 to 2019 can only be compared with the three previous waves to a limited extent, so that trends over time can only be made with caution. Whether and to what extent the gender gap in volunteering and the assumption of leadership positions can be closed can only be assessed through further survey waves. Furthermore, it must be conceded that the current waves of the German Survey on Volunteering do not provide any further information on how the respondents feel involved in the respective organizations/associations, how much participation is made possible, etc. Association studies, however, indicate positive correlations between the satisfaction of volunteers and their commitment (Schlesinger et al., 2014). In addition to individual incentive and expectation structures, organizational factors such as involvement in the club, club memberships of one's own children, the perceived club climate, staff management, the reputation of the club, or the accumulated human and social capital obviously play a role in whether, to what extent and in what form members volunteer in the sports club (Flatau, 2009; Dwyer et al., 2013; Schlesinger et al., 2013, 2014; Egli et al., 2014; Malinen and Harju, 2017; Swierzy et al., 2018; Wicker et al., 2018). As a third level, contextual factors such as political, social or economic conditions should also be considered to explain individual volunteering and related gender differences in the context of a multilevel analysis (Bühlmann and Freitag, 2004, 2007). These analyses should not rely on quantitative methods alone, but should integrate qualitative methods in order to explain statistical findings comprehensively (Kelle, 2007) and to understand the mechanisms of reproduction of social inequality.

Another limitation of our data is the cross-sectional design of the survey. In the future, more panel data should be collected so that causal relationships can be tested. This is because variables often correlate with others, and the complex relationships can be interpreted in both causal directions (Einolf and Chambré, 2011). For example, we operationalized indicators of social networks and health as explanatory variables of volunteering. However, they can also be conceptualized as an effect of volunteering (for an overview see Wicker, 2017; Wicker and Downward, 2019).

The German Survey on Volunteering is mainly used for (sports) political reporting. Up to now, it lacked a theoretical basis. With Solga's model, we have attempted to conceptualize volunteering as a dimension of social inequality. We investigated the question of which determinants cause unequal access to voluntary engagement and to leadership positions in sport. Gender differences and interactions between gender and other determinants of social inequality were of particular interest. Mechanisms of reproduction of social inequality and the effects of unequal access to volunteering or leadership positions in sports clubs were only plausibilized in the paper. This needs to be addressed in future studies.

We believe, however, that the findings provide a basis for deriving more targeted strategies for attracting (and retaining) women in volunteer activities and especially in leadership positions. The benefits of greater gender diversity in leadership positions have already been highlighted in various studies (for an overview see Wicker et al., 2020). Our findings suggest that it is not enough for family members and friends to motivate women to take up leadership positions. Recruitment should increasingly come from leaders in sports clubs and organizations or even employers. The latter is more common among men. It could also encourage women if sports clubs and organizations clearly communicated the necessary qualifications that women often have when filling vacancies and also pointed out which social skills can be acquired through voluntary work. However, many barriers, such as the aforementioned time problems, cannot be solved by the clubs. Moreover, in terms of public policy, there seems to be a need, for example through tax incentives and improved childcare, to distribute gainful, family and voluntary work more equitably between the sexes. For women, it is crucial that voluntary work does not start only after the family phase, when it is often too late for a career that leads to a leading position in voluntary work. An early reduction in working hours would enable men to contribute more to domestic and family work and could lead to women working part-time having more time not only for gainful employment, but also for developing a career in voluntary work and in the end gain a leadership position.

## Data Availability Statement

Data are from the public release of the German Survey on Volunteering (FWS), provided by the Research Data Centre of the German Centre of Gerontology (FDZ-DZA). FWS microdata are available free of charge to scientific researchers for non-profitable purposes. The data is available at the Research Data Centre of the DZA: https://www.dza.de/en/research/fdz/german-survey-on-volunteering/fws-data-access.

## Ethics Statement

Ethical review and approval was not required for the study in accordance with local legislation and institutional requirements. The participants gave their consent to participate in this study by telephone. Further explanations can be found in the method reports on the German Volunteer Survey (Schiel et al., 2020).

## Author Contributions

UB conceived and designed the paper and wrote the first draft of the paper. UB and SS analyzed the secondary data and approved the submitted manuscript. SS provided substantial edits to the paper. Both authors contributed to the article and approved the submitted version.

## Funding

The project Voluntary Engagement in Sport: Sport-Related Special Evaluation of the German Survey on Volunteer (2014 and 2019) received a research grant from the Federal Institute for Sport Science based on a resolution of the German Bundestag. The article processing charge was funded by the Deutsche Forschungsgemeinschaft (DFG, German Research Foundation) - 491192747 and the Open Access Publication Fund of Humboldt-Universität zu Berlin.

## Conflict of Interest

The authors declare that the research was conducted in the absence of any commercial or financial relationships that could be construed as a potential conflict of interest.

## Publisher's Note

All claims expressed in this article are solely those of the authors and do not necessarily represent those of their affiliated organizations, or those of the publisher, the editors and the reviewers. Any product that may be evaluated in this article, or claim that may be made by its manufacturer, is not guaranteed or endorsed by the publisher.

## Footnotes

^1^However, volunteering can also be seen as a determinant of social inequality (e.g., signal functions of volunteering in the application procedure) or as an effect or result of other inequality dimensions (e.g., spill-over effect).
